# Magnetic and Magnetostrictive Properties of Ni50Mn20Ga27Cu3 Rapidly Quenched Ribbons

**DOI:** 10.3390/ma14185126

**Published:** 2021-09-07

**Authors:** Mihaela Sofronie, Mugurel Tolea, Bogdan Popescu, Monica Enculescu, Felicia Tolea

**Affiliations:** National Institute of Materials Physics, Atomistilor 405A, 077125 Magurele, Romania; mihsof@infim.ro (M.S.); tzolea@infim.ro (M.T.); bogdan.popescu@infim.ro (B.P.); mdatcu@infim.ro (M.E.)

**Keywords:** ferromagnetic shape memory alloys, martensitic transformation, premartensitic transformation, rapid solidification, magnetostriction, magnetic properties, magnetoresistance

## Abstract

The influence of the rapid solidification technique and heat treatment on the martensitic transformation, magnetic properties, thermo- and magnetic induced strain and electrical resistivity is investigated for the Cu doped NiMnGa Heusler-based ferromagnetic shape memory ribbons. The martensitic transformation temperatures are unexpectedly low (below 90 K—which can be attributed to the disordered texture as well as to the uncertainty in the elements substituted by the Cu), preceded by a premartensitic transformation (starting at around 190 K). A thermal treatment slightly increases the transformation as well as the Curie temperatures. Additionally, the thermal treatment promotes a higher magnetization value of the austenite phase and a lower one in the martensite. The shift of the martensitic transformation temperatures induced by the applied magnetic field, quantified from thermo-magnetic and thermo-magnetic induced strain measurements, is measured to have a positive value of about 1 K/T, and is then used to calculate the transformation entropy of the ribbons. The magnetostriction measurements suggest a rotational mechanism in low fields for the thermal treated samples and a saturation tendency at higher magnetic fields, except for the temperatures close to the phase transition temperatures (saturation is not reached at 5 T), where a linear volume magnetostriction cannot be ruled out. Resistivity and magnetoresistance properties have also been measured for all the samples.

## 1. Introduction

Specific to the shape memory alloys (SMAs), the so-called martensitic transformation (MT), is a thermo-elastic reversible structural phase transition between a high symmetry phase and a lower one (see, e.g., [1]). On cooling, the high-temperature austenite phase undergoes a diffusionless transformation in which atoms shift cooperatively, reducing the symmetry and forming the low-temperature martensite phase. Ferromagnetic SMAs (FSMAs) are materials with an MT temperature lower than the magnetic transition temperature. For Heusler type FSMAs, the transition takes place between austenite (with B2 or ordered L21 structure) and either a seven-layer (14 M), a five-layer modulated (10 M), or a non-modulated (L10 tetragonal) martensite structure, depending on the composition and thermal history (see, e.g., the recent paper [2] and references therein).

In these compounds, the coupling of the structural and magnetic degrees of freedom may be able to trigger a rearrangement of the martensitic variants under the applied magnetic field promoting the magnetic field induced strains (MFISs). The large values of MFISs, the high-frequency response and the shape memory effect evidenced in FSMAs recommend them as promising materials for magnetically controlled actuators. A large MFIS of about 0.2% was first reported for Ni-Mn-Ga monocrystals [3] and later up to 5.1 and 9.5% were reported for the five-layered [4] and seven-layered martensite phase [5], respectively. The magnetic induced strain has much lower values in polycrystalline Ni-Mn-Ga (tens of ppms) [6,7], and the values are not improved by the thermal treatments, even if they do improve the austenite to martensite transition (a clearer peek in calorimetry).

Furthermore, in addition to different substitutions [8,9], the rapid quenching from the liquid state by the melt spinning technique offers one possibility to obtain almost-ready shaped material (ribbons) with improved ductility [8]. Rapidly quenched ribbons with tailored MT and Curie temperatures may offer new opportunities for applications as miniaturized active elements for sensors, actuators and other functional devices.

Previous studies indicate that the partial Cu-doping of Ni-Mn-Ga as bulk [9,10,11] or ribbons [12,13] allows for the smooth adjustment of the magnetic and structural transition temperatures. The first principles calculations suggest that, if present in a small amount, the Cu atoms prefer taking the sublattice of the host element in deficiency [14]. Further ab-initio studies suggest that the partial, and even the complete, substitution of Mn by Cu lead to more stable compounds compared to those in which Cu replaces Ni or Ga [15].

In this paper, we study the structural, transport, magnetic and magnetostrictive properties of Ni2MnGa alloys, which are Ga-rich and with Cu added in a small amount, prepared as ribbons by rapid solidification. 

## 2. Material and Methods/Experimental

The polycrystalline Ni50Mn20Ga27Cu3 sample was prepared from high-purity elements by arc melting in an argon atmosphere. The resulted ingot was inductively melted in a quartz tube under an argon atmosphere and then rapidly quenched using the melt spinning technique. As-prepared ribbons (denoted Mn-AP) of about 5–6 cm length, 2 mm width and 25–38 μm thickness were obtained by evacuating the melt on a rotating copper wheel (linear velocity of 20 m/s) through a quartz crucible with a linear nozzle (0.5 mm). Subsequently, the ribbons were annealed at 673 K for 20 min (TT), followed by slow cooling (denoted Mn-TT). The degree of L21 atomic order is low for the rapidly quenched Mn-AP, and the temperature of the thermal treatment was chosen to be sufficient high to allow the migration of atoms, but still below the B2–L21 transition (see [16]). 

Since our samples have low martensitic transformation temperatures, the differential scanning calorimetry (DSC), by using the Netzsch Differential Scanning Calorimeter (working in the temperature range from 90 up to 873 K), does not detect the phase transition. As such, an additional evaluation was realized by the thermo-magnetic measurements using the MPMS-SQUID-QD magnetometer in Reciprocal Space Option (RSO mode with the temperature range from 4 up to 390 K and magnetic field up to 9 T), with the magnetic field along ribbon length (the rolling direction) to minimize the demagnetization field effect. Thus, on the cooling/heating sequence under a small magnetic field (0.02 T), the thermo-magnetic hysteresis, an indicator of the MT, was detected and allows its characteristic temperatures to be obtained. By using the same magnetometer, the magnetic properties were evaluated. The structural investigations were conducted using X-ray diffraction (XRD), using a Bruker D8 Advantage diffractometer in the Bragg-Brentano geometry, with Cu K X-ray source, operated at 40 kV, 40 mA, at room temperature (RT). The microstructure was observed using the Scanning Electron Microscopy (SEM) via a Zeiss Evo 50 XVP microscope, and the ribbons compositions were checked using Energy Dispersive X-Ray Spectroscopy (EDS) employing a Zeiss Evo 50 XVP microscope equipped with a Bruker EDS detector.

The magnetostrictive measurements were achieved by using the strain gauges method. This method used two strain gauges (1-LY11-1.5/120, HBM), one being cemented (with Z70—cold curing superglue from HBM) on ribbon length and the other serving as reference (to compensate for the thermal and magnetoresistive effects of the strain gauge). The “Vishay Micro-Measurements Model P3 strain indicator and recorder” and the magnetic platform Cryogenic Ltd. were used for fulfilling this study. In-plane strains (along the length of the ribbon) were recorded with a different magnetic field applied parallel (parallel with sample length) and transverse (perpendicular to the sample thickness) at constant or different temperatures (the temperature range from 30 up to 300 K and fields up to 3 T).

The temperature and magnetic field dependences of the electrical resistivity were recorded in order to characterize the electrical transport, and to evaluate the magnetoresistive effect on the samples through the premartensitic and martensitic transformations. These investigations were performed in standard four-probe method (with contacts made with silver paste) using a Quantum Design Physical Property Measurement System (QD-PPMS). The measurements have been performed with the current along the longitudinal direction of the ribbons and the magnetic field perpendicular to the ribbons. 

## 3. Results and Discussion

### 3.1. XRD Results

**Figure 1:**
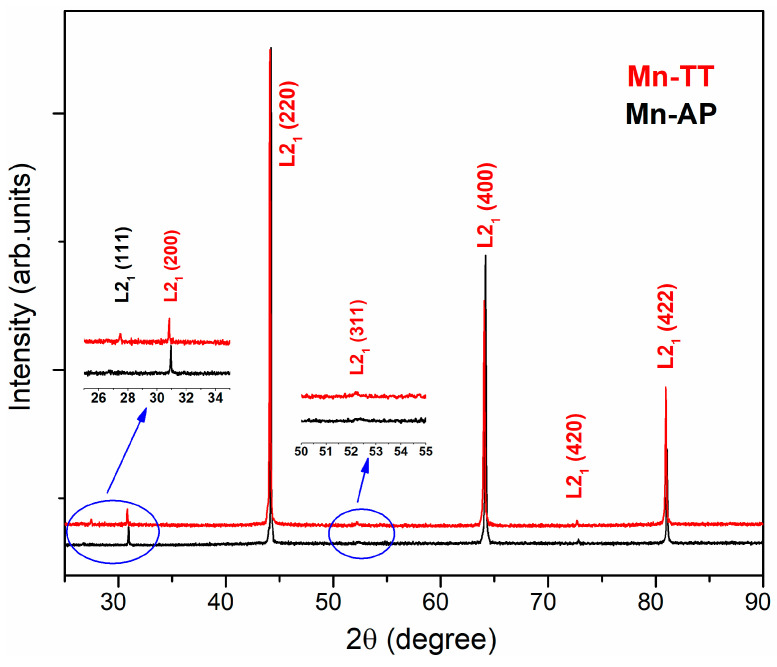
X-ray diffraction patterns recorded on the CS for the as-prepared (Mn-AP) and thermally treated (Mn-TT) ribbons at room temperature.

The X-ray diffraction (XRD) patterns recorded on the CS at room temperature for the as-prepared (Mn-AP) and thermally treated (Mn-TT) ribbons are shown in Figure 1. The as-prepared ribbon exhibits the B2 disorder phase at room temperature, while, after thermal treatment, the (111) and (311) peaks with noticeable intensity appear (see the Insets in Figure 1), an indicator that the crystal structure changes to the ordered L21 phase. Kreissl et al. [17] report that the degree of the atomic order of the L21 structure plays a significant role in determining the martensitic transition temperature.

Therefore, the disordered B2 structure suppresses the MT at 100 K from the stoichiometric alloy, with a strong increase at 200 K for the L21 ordered structure (a = 5.855 Å). The slight contraction of the lattice parameter (a = 5.809 Å) is observed in our sample following the partial replacement of Mn with Cu, due to the fact that the covalent radius of Mn (rMn = 1.39 Å) is slightly larger than that of Cu (rCu = 1.32 Å). As expected, the ribbons are highly textured, as the enhanced intensity of the (400) reflection suggests, giving rise to a columnar microstructure. This texture is the result of the different cooling velocity of the contact side (with the wheel surface) and the free side of the ribbons [18,19,20].

### 3.2. SEM Microscopy

**Figure 2:**
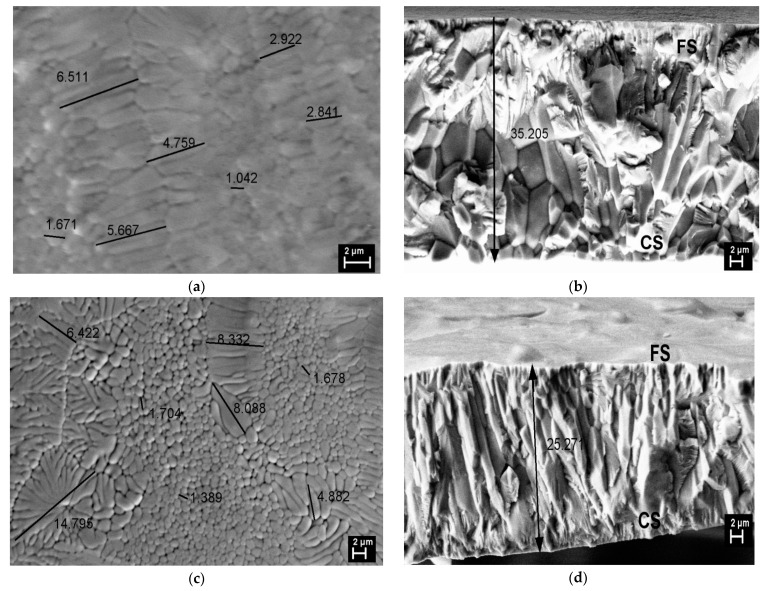
SEM images on FS and the fractured cross-section for Mn-AP (**a**,**b**) and Mn-TT (**c**,**d**).

Figure 2 shows the scanning electron microscopy (SEM) analysis on the FS surface for the as-prepared (Mn-AP) and the thermally treated sample (Mn-TT) and their cross-section morphology at RT.

For the Mn-AP ribbons (Figure 2a), the SEM image shows a mixture of cellular and dendritic structures, with small grain sizes between 1–6.5 μm without the specific martensitic relief. The substantial grain refinement is an effect of rapid quenching at a high wheel velocity [21]. The dendritic structure is also a result of the rapid solidification and the high temperature gradient between liquidus and solidus, specific to the off-stoichiometric Ni-Mn-Ga alloys [22]. Figure 2b, on the fractured cross-section, indicates the equiaxial and columnar grains from the contact surface (CS) with the copper wheel to the free surface (FS). The presence of the columnar grains is a microstructure signature of the fast nucleation and growth process along the cross-section during the rapid cooling of the melt due to the temperature gradient typical for the melt-spinning technique [23]. Rama-Rao et al. [22] claim that the microstructure of ribbons gradually changes during the processing route to the dendritic structure when a concentration variation of the elements appears, especially in Ni. The SEM image on the Mn-TT surface indicates the increase in the cellular and dendritic grain size (the last ones up to 15 μm) due to the stress release and defects density reduction (Figure 2c). The fractured cross-section structure shows that the morphology changes with TT, being a noticeable improvement of the columnar microstructure without equiaxial grains or precipitates (Figure 2d). According to the previous studies performed by our group [24,25], and also as suggested by XRD (Figure 1), the treatment temperature of 400 °C (673 K) is enough to promote the ordering of the structure and the growth of crystallites. We also mentioned that, since the thermal treatment is not performed in situ at SEM, different parts of the samples are imaged before and after annealing.

The ribbons chemical composition results are listed in Table 1, as measured using EDS (within the limits of the method accuracy).

The variation in concentrations from Table 1 may originate both from a non-uniform distribution of elements on the surface of the as-prepared ribbons or a migration of elements during thermal treatment. We mention that studies suggest [26] that the ability of Cu to strengthen grain boundaries and its increasing concentration prevents the rapid growth in the grain size of the cellular structure compared to the dendritic one.

### 3.3. Magnetic Behavior

**Figure 3:**
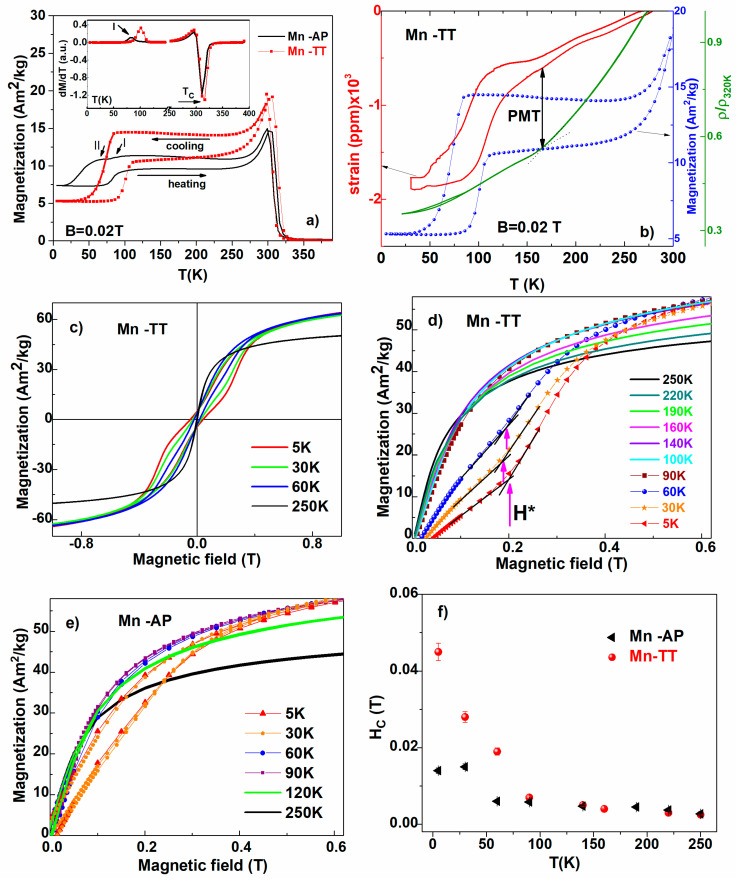
(**a**) The temperature dependence of magnetization on Mn-AP and Mn-TT ribbons at low temperature in 0.02 T applied magnetic field; Inset: The first derivative of magnetization dM/dT; (**b**) The thermo-magnetic, thermo-resistivity and thermal expansion measurements to identify MT interval and also the value of the PMT start temperature (at 190 K) for the Mn-TT sample; (**c**) Hysteresis loops for Mn-TT ribbons; (**d**) isothermal dependences of magnetization versus applied field for Mn-AP sample. The critical field H* is 0.29 T at 5 K and 0.22 T at 60 K; (**e**) magnetization dependences on the applied field for Mn-TT ribbons. (**f**) The temperature dependence of the coercive field.

The thermomagnetic measurements at low temperatures, under an applied magnetic field (0.02 T), reveal a thermal hysteresis during the cooling/heating sequences, which is a signature of the martensitic transformation. The magnetization jumps (Figure 3a) on the cooling/heating curves allow of the MT characteristic temperatures to be evaluated by using the tangential method, and they are listed in Table 2. The as-prepared sample has a wide range thermal hysteresis, which significantly reduces (~15 K) after the heat treatment. This wide thermal hysteresis may be related to a wide distribution of elastic stress in the dentritic/cellular structures of the as-prepared sample. It is also worth mentioning that the Cu substitution induces a strengthening of the grain boundaries; therefore, the total elastic energy decreases overall [26]. Moreover, a small jump of magnetization (at 87 K denoted I in Figure 3a) before the highest ones (at 55 K denoted II in Figure 3a) is observed only on the cooling curve of the Mn-AP sample, which indicates the existence of a two-step transformation, possibly due to the premartensitic transformation. It is important to note that the martensite start temperature (Ms) for the Mn-TT sample coincides with that of the MT first step of the Mn-AP. The transformation temperatures are reduced due to the disorder of small-grained, textured and stressed samples and to the uncertainty of Cu substituted elements.

At cooling, the abrupt increase in the magnetization near 310 K for Mn-AP and 316 K for Mn-TT mark the magnetic order–disorder transition Tc (see Inset of Figure 3a), next to the Hopkinson peak specific to the Ni-Mn-Ga ribbons. For the near stoichiometric Ni2MnGa [22,23,27] and in the Cu-doped NiFeGa [25,28] ribbons rapidly quenched or annealed at low temperatures, a similar behavior has been reported. The Hopkinson peak was observed for first time in iron (soft magnetic material) and its origin was associated with the magneto-anisotropy constant reduction with the temperature increases up to the Curie point [29].

For Heusler type FSMAs, the magnetization decreases between Tc and Ms temperatures during the premartensitic transformation (PMT) as a result of the magnetic anisotropy enhancing [30,31]. The thermo-magnetic, thermal resistivity and thermal expansion measurements to identify MT and the existence of the pre-martensitic transformation (indicated with arrow) have been used for the Mn-TT sample (Figure 3b) and Mn-AP (not shown). The lower values of the martensitic transformation and Curie temperatures are explained by the high degree of quenched-in short-range disorder for Mn-AP and recovered for Mn-TT upon heat treatment due to the improvement in the ordering by a vacancy migration mechanism and the increase in the grain’s size [30].

More comprehensive data have been obtained from the M(H) curves that achieved up to 4 T at different temperatures during cooling from 250 to 5 K (Figure 3c shows up to 1 T). All the measurements have been realized with the magnetic field parallel to the length of the ribbon. The magnetization values (in austenite and martensite phase) show a slight increase after the thermal treatment (Figure 3d,e), without saturation up to 4 T. The hysteresis loops in martensite show the so-called wasp-waist shape for the Mn-TT ribbons, and this is missing for the Mn-AP sample with a weak ferromagnetic behavior (in Figure 3e). When the temperature decreases, the critical fields denoted with H* (defined by the inflexion point, see Figure 3d) shift to higher magnetic field values, e.g., the critical field H* is 0.29 T at 5 K and 0.22 T at 60 K for the Mn-TT sample, similar to the reported Ni-Mn-Ga ribbons [22,32]. The magnetic variants’ reorganization correlates with the high magneto-crystalline anisotropy of the martensite and the high mobility of the twin boundaries assigned this behavior. The process is reversible due to the grain-to-grain elastic energy stored in the polycrystalline ribbon release after removing the magnetic field [22,32,33]. The coercive field (Hc) increases monotonously up to Ms on cooling from temperatures below TC for the Mn-based ribbons (Figure 3f). The pre-martensitic transformation slightly enhances the magnetocrystalline anisotropy and increases the coercive field [30]. The last one has a monotonous increase for the as-prepared sample and rapidly for the thermally treated ones in the martensite state. The highest value for Hc (~0.045 T) is obtained at low temperatures for the Mn-TT ribbons, in agreement with the thermomagnetic data.

Evidence of a premartensitic phase, which forms from austenite on cooling and transform in martensite at lower temperatures, can be found in Figure 3b. Similar to the martensitic transformation, the premartensitic one is also a first order transition [34,35] and does show a hysteresis (seen in the magnetization and strain curves). Additionally, the premartensite start temperature corresponds to the slope change in resistivity (at about 190 K) and to a small variation of the strain. The premartensitic transformation takes place on a wider interval than the martensitic one.

### 3.4. Magnetoelastic Properties/Magnetostriction

**Figure 4:**
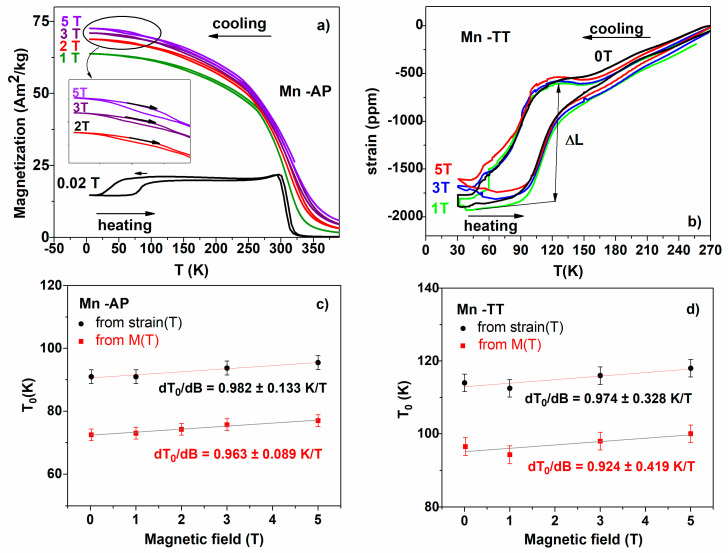
(**a**) Iso-field magnetization curves performed by cooling–heating the ribbons; (**b**) thermo-strain curves recorded at 0, 1, 3 and 5 T applied magnetic field for Mn-TT; (**c**) The T_0_ dependences as a function of the applied magnetic field from thermomagnetic and thermo-strain measurements for Mn-AP; (**d**) the same for Mn-TT samples.

Thermomagnetic measurements were performed under different constant fields (0.02–5 T) by cooling down the samples (from 390 to 5 K), and warming them up again, in order to evidence the influence of the magnetic field intensity on the MT temperatures (see Figure 4a for Mn-AP). When cooling without an applied magnetic field, can be seen a continuous contraction passing through the MT (Figure 4b). The martensite variants can accommodate the strain to minimize the elastic energy and keep the shape of the ribbons (see [36] for a model based on different plates sizes).

In Ni-Mn-Ga alloys, the magnetoelastic coupling is described by a negative magnetostriction constant (see, e.g., [32]) and can be correlated with the appearance of an easy-magnetization direction along the short c-axis accompanied by the large uniaxial magnetic anisotropy [33]. By heating, the strains are completely recovered via expansion, and the ribbons show a good thermo-elastic transformation with extended thermal hysteresis. Under the applied magnetic field starting from the austenite state and then cooling and passing through MT, the martensite variants nucleate and grow preferentially along the field direction to minimize the Zeeman energy. The nucleation mechanism is influenced by the grain size and the cracks within them or at their boundaries, being less dependent on the mobility of the martensitic variants [37].

The behavior evidenced in the thermomagnetic data have correspondence in the thermo-strain curves, which were performed both without an applied magnetic field and with an applied field (up to 5 T), in the temperature range of interest for our samples (shown in Figure 4b for Mn-TT). These allowed us to indicate the temperatures characteristic of premartensitic and martensitic transformations and determine the influence of the magnetic field on them. The pre-martensitic temperature variation with the applied magnetic field, indicated as 190 K at 0 T, is not visible on the thermo-strain dependence curves. The martensite start (Ms) and austenite finish (Af) temperatures were evaluated using the tangential method. The temperatures determined on the thermo-strain curves similar to those on the thermomagnetic curves have higher values (~15 K). The possible cause could be the strain measurement method that involves the direct contact of the strain gauge glued to the sample. For a quantitative evaluation of the shift, the thermodynamic MT temperature under the applied magnetic induction B, T_0_(B) = (Ms + Af)/2, was determined for each value of the applied magnetic field. The dependences of T_0_(B) as a function of the applied magnetic field from thermomagnetic and thermo-strain measurements are plotted in Figure 4c,d for the Mn-AP and Mn-TT samples. The equilibrium MT temperatures increase linearly with the magnetic field induction, and the slopes (0.963 ± 0.089 K/T for Mn-AP and 0.924 ± 0.419 K/T for Mn-TT), as resulted from the linear fit of the thermomagnetic data, are confirmed by the thermo-strain ones (0.982 ± 0.133 K/T for Mn-AP and 0.974 ± 0.328 K/T). 

Sakon et al. [38] indicate, for the shift of the MT temperature, a close value (1–1.1 K/T) in bulk NiMnGa alloys with a Cu substitution. The influence of the external magnetic field on the transformation temperatures can also be discussed using the following Clausius–Clapeyron equation: dT/dB = −ΔM/ΔS, where ΔM is the difference in magnetization between austenite and martensite and ΔS is the entropy change associated with the transformation (evaluate from calorimetric measurements). In our case, due to the low values of the transformation temperatures, the direct calorimetric measurements of ΔS are not accessible (as conducted in, e.g., [39]); therefore, the values of ΔS have to be calculated from the Clausius-Clapeyron equation. By using this method for the thermomagnetic measurements during heating in 3 T, it was obtained that ΔM = −0.788 Am^2^/kg for the Mn-AP sample and ΔM = −2.867 Am^2^/kg for the Mn-TT sample. The resulting values for entropy variation (ΔS = 2.163 J/(kg·K) for Mn-AP and ΔS = 6.414 J/(kg·K) for Mn-TT) are small compared with those indicated for the bulk alloys with a Cu substitution.

**Figure 5:**
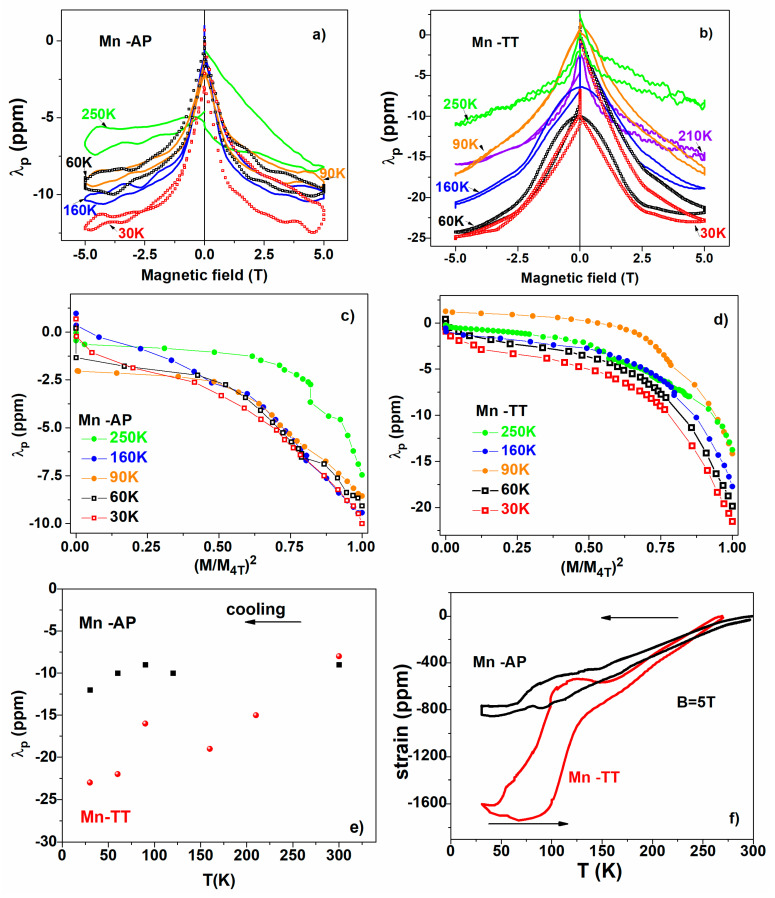
(**a**) Isothermal magnetostriction measurements performed at different temperatures in magnetic fields up to 5 T, applied parallel to the measuring direction for Mn-AP; (**b**) the same for Mn-TT ribbons; (**c**) the parallel magnetostriction plotted versus the square of (M/M4 T) for Mn-AP; (**d**) the same for Mn-TT ribbons; (**e**) the temperature dependence of parallel magnetostriction (λp); (**f**) thermo-strain dependencies recorded in 5 T.

Isothermal magnetostriction measurements in magnetic fields up to 5 T, applied parallel to the measuring direction, were performed at different temperatures, specific to each sample. The values of the magnetic field induced strain are smaller, but of the same order of magnitude as reported in [32] for polycrystalline Ni2MnGa, also having the same negative sign. The performed thermal treatment leads to a slight increase in the field induced strain (See Figure 5a,b).

Figure 5c,d show the parallel magnetostriction plotted versus the square of (M/M4 T). Usually, the saturation magnetization is used, but we used the magnetization at 4 T since saturation is not reached. At low fields, the linear dependence (clearer for the Mn-TT) confirms a pure rotational mechanism. At higher fields, the more abrupt decrease (both for austenite and martensite phases) is due to the magnetization saturating faster than the magnetostriction.

The volume magnetostriction mechanism cannot be ruled out for the Mn-TT samples close to the transformation temperatures (see the linear tendency of the orange and blue curves—90 and 160 K—in Figure 5b, for fields higher than 2 T, away from the rotational parabolic regime), but is not plausible for the other curves, which show saturation tendencies.

The quadratic regime at low fields (see Figure 5a) allows, in principle, the employment of a phenomenological model describing the spin rotation mechanism [40,41], which expresses the strain in terms of magnetoelastic coupling and the shear constant (C’) (see Equation (6) from [41]). In the case of bulk Ni2MnGa, the elastic shear constant C’ has very low values (2.5 GPa in austenite and 89 GPa in martensite [42]), which favor the large magnetic field induced strain by the re-orientation of variants. However, the constants for the polycrystalline Ni2MnGa are expected to differ from those of the bulk [6], and also the field induced strain is much less. The re-orientation of variants may still play a role, but the overall effect is much reduced due to the orientation randomness [6]. The temperature dependence of the parallel magnetostriction (λp) shown in Figure 5e follows the same trend as the temperature dependence of the total strain (Figure 5f).

### 3.5. Transport Properties

**Figure 6:**
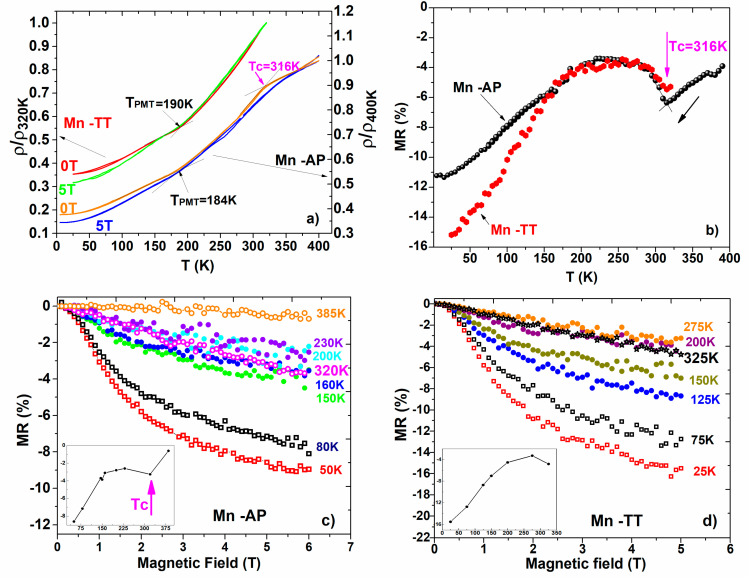
(**a**) Reduced resistivity versus temperature in 0 and 5 T; (**b**) evaluation of MR% from temperature dependence of Ac resistivity (*ρ*) for samples Mn-AP and Mn-TT; (**c**) resistivity change under applied magnetic field at different temperatures for Mn-AP and (**d**) for Mn-TT samples. Insets: shows the temperature dependence of MR% at 5 T field obtained from MR(H) dependency.

The temperature dependence of the reduced resistivity measured without and with an applied magnetic field (5 T) for both the studied samples are presented in Figure 6a. For both the as-prepared and thermal treated samples, the resistivity shows slope changes corresponding to the transformation between the austenite and the premartensitic phase (PMT) at T_PMT_ and between the PMT phase and the martensitic phase at lower T_MT_ temperatures. The PMT preceding the formation of the martensite was also evidenced by magnetic measurements and strain evolution (Figure 3b) but was most clearly seen in the resistivity measurements *ρ*(T), as reported also by [43,44] for Ni-Mn-Ga polycrystalline ingots, and in [31] for Ni2MnGa ribbons.

It is important to notice that the thermal hysteresis in resistivity is either absent or small. The thermal treated Mn-TT sample, with slightly higher transformation temperatures, shows a small hysteresis between 95 and 44 K (in 0 T), and 87 and 50 K (in 5 T).

The low temperature dependence of resistivity (shown in Figure 6a) reveals a metallic behavior for Mn-AP and Mn-TT samples: the resistivity decreases on cooling due to the phonon scattering [45], with slope changes at the PMT and MT. At 315 K, the Curie temperatures from both *ρ*(T) and MR(T) are revealed (Figure 6a–d) and the values coincide with the ones previously obtained from the magnetic measurements. Specifically for the SMAs, the resistivity decreases when the magnetic field is applied [43,46], and our NiMnCuGa ribbons show the same behavior for both the AP and TT samples (Figure 6a,b).

We remind the reader of the contributions to *ρ*(T): the lattice contribution (electron–phonon scattering) and the magnetic contribution (electron–magnon scattering), with different temperature dependencies. Their effects are considered to be small. There is also residual resistivity (a temperature independent contribution) related at the lattice defects and chemical disorder, whose role is often underestimated; Barandiaran et al. [43] suggests that the main term affecting the resistivity variations at the transition is the change in the residual resistivity due to the increase in the static disorder.

The magnetoresistive effect (MR%) of magnetic alloys reflects the resistivity change due to the reduction in the magnetic disorder caused by an applied magnetic field at a given temperature. It was evaluated using the following formula:

(1) $$\mathrm{MR}=\frac{\rho \left(H\right)-\rho \left(0\right)}{\rho \left(0\right)}$$

where *ρ*(0) and *ρ*(H) are the values of the resistivity in zero and 5 T magnetic applied fields, for both the Mn-AP and Mn-TT samples—shown in Figure 6b. To further characterize the MR%, the continuous variation of the resistivity with the magnetic field at different fixed temperatures, corresponding to the austenitic phase, to the PMT, and also the martensitic phase, was measured (Figure 6c,d).

Since we have a ferromagnetic material, one finds, as expected, a negative MR increasing with temperature and having a peak at Tc. The existing literature on the MR behavior of FSMAs report a wide class of properties, influenced not by the stoichiometry but also by the preparation route, thermal treatments, etc. [23,27,31,43,44,46,47,48,49,50,51]. While the NiFeGa-based alloys show a higher magnetoresistive effect for the austenite than for the martensite [43,47,51], our Cu-doped Ni-Mn-Ga alloys show a different behavior. For both samples (Mn-Ap and Mn-TT), after a broad negative maximum at the Curie temperature (Figure 6b), the MR% keeps a quasi-constant value until the T_PMT_ is reached (indicated as an inflexion point in the *ρ*(T) curves in Figure 6a) and then starts to grow (negative values) as the temperature decreases. One can notice that the resistivity change under an applied magnetic field evaluated at different temperatures also reveal a higher MR% in martensite than in austenite (see also Figure 6c,d).

The premartensite shows a peculiar behavior of MR%, as previous papers also report [42] for the polycrystalline Ni51.1Mn24.9Ga24 alloy. According to [42], for Cu-doped Ni-Mn-Ga Heusler alloys, the MR% behavior can be explained by an intrinsic magnetic scattering mechanism, due to the magnetic inhomogeneities.

## 4. Conclusions

Ribbons with the Ni50Mn20Ga27Cu3 nominal composition have been prepared by melt-spinning and characterized using different methods, both as-quenched and after a thermal treatment (20 min at 673 K). The preparation route induces a mixture of cellular and dendritic structures on the surface, with grain sizes of a few μm, having a columnar aspect in the cross-section. The thermal treatment favors the growth of the grains and dendritic structures, a reduction in the defects and a promotion of the ordered L21 phase (evidenced using XRD).

The thermo-magnetic, strain and resistivity measurements confirm a martensitic transformation at low temperatures (below 90 K) preceded by a premartensite transformation (starting at around 190 K). The thermal treatment increases the transformation temperatures at about 20 K. Such low transformation temperatures can be attributed to the atomic disorder and an uncertainty in the elements substituted by Cu. The Curie temperatures are also low (310 K, for the austenite phase) and increase slightly after thermal treatment (316 K). The indications that there is a premartensitic phase—formed on cooling from austenite, before the martensite phase is stabilized—are seen in the magnetic measurements, the strain measurements and in the resistivity measurements (both for the Mn-AP and Mn-TT samples).

The shift of the martensitic transformation temperatures induced by an applied magnetic field has positive values of about 1 K/T, which, correlated with the measured magnetization jump, allowed us to extract the entropy variation via the Clausius-Clapeyron equation.

The magnetostriction has negative values (of about 10–20 ppm in 5 T), smaller than but comparable with previously reported data on the undoped polycrystalline Ni2MnGa. For the thermal treated samples, the parabolic dependence of λp at low fields suggest a spin rotational mechanism, while, at high fields, a saturation tendency can be noticed, except for the temperatures close to the transformation, where a saturation is not noticed and a volume magnetostriction can be assumed.

The Mn-AP and Mn-TT ribbons show a maximum in the MR% at the Tc, a quasi-constant behavior before the premartensitic transformation and then the specific behavior for a ferromagnet, increasing (in absolute value) with a decreasing temperature. 

## Figures and Tables

**Table 1:** The chemical composition for as-prepared (Mn-AP) and thermally treated (Mn-TT) ribbons.

Sample	Ni (at.%)	Mn (at.%)	Ga (at.%)	Cu (at.%)
**Mn-AP**	49.9	21.0	25.1	3.9
**Mn-TT**	51.3	19.8	24.7	4.2

**Table 2:** The characteristic temperatures—martensite start (Ms) and finish (Mf), austenite start (As) and finish, (Af)—the range of martensitic transformation (Af-Mf), the thermal hysteresis Af-Ms the Curie temperatures (Tc) for as-prepared and after TT samples determined from the thermo-magnetic measurements. The thermodynamic equilibrium temperature T_0_ is definite as T_0_ = (Ms + Af)/2.

Sample	Ms//Mf (K)	As//Af (K)	T_0_ (K)	Af-Ms (K)	Af-Mf (K)	T_C_ (K)
Mn-AP	(I)88-(II) 56//24	76//94	75	38	70	310
Mn-TT	86//53	91//108	97	22	55	316

## Data Availability

Data is available, upon reasonable request, addressed to the corresponding author.
